# The gut microbiota features and the application value in predicting recurrent risks for gallstone patients who underwent laparoscopic cholecystectomy

**DOI:** 10.1128/msystems.01760-24

**Published:** 2025-07-25

**Authors:** Huiqiang Li, Weili Li, Xinzheng Liu, Jianhua Zhang, Chuncheng Lin, Shushen Ji, Huihui Jiang, Tao Wang, Zijian Su

**Affiliations:** 1 Department of Hepatobiliary Surgery, Quanzhou First Hospital 117894 , Quanzhou, Fujian, China; 2 Zhangjiang Center for Translational Medicine, Shanghai Biotecan Pharmaceuticals Co., Ltd., Shanghai, China; 3 Department of Clinical Laboratory, Huanghe Sanmenxia Hospital, Sanmenxia, Henan Province, China; 4 Department of General Surgery II, Huanghe Sanmenxia Hospital, Sanmenxia, Henan Province, China; University of California, San Francisco, San Francisco, California, USA

**Keywords:** gallstones, gastrointestinal microbiome, laparoscopic cholecystectomy, choledocholithiasis

## Abstract

Gut microbiota is associated with gallstone occurrence and recurrence. But whether they can predict post-cholecystectomy choledocholithiasis recurrence needs to be investigated. From September 2020 to June 2021, a total of 100 symptomatic gallstone patients scheduled for laparoscopic cholecystectomy were enrolled in the Disease group in the Department of Hepatobiliary Surgery at Quanzhou First Hospital. Meanwhile, a total of 50 age- and sex-matched healthy controls were included in the Control group. Fecal specimens were collected from both groups and subjected to 16S rDNA sequencing (V3V4 region) for microbiologic analysis. After laparoscopic cholecystectomy, patients were followed up, and recurrent cases were recorded. Finally, a nomogram for predicting recurrent risks was built. The gut microbial diversity in the Disease group was significantly lower than that of the Control group (all *P* < 0.05). The Chao1 index of the recurrent group was remarkably lower than the control group (*P* < 0.05). Linear discriminant analysis (LDA) effect size (LEfSe) analysis showed that *Phocaeicola dorei* (LDA score = 4.2, *P* < 0.05) was the feature microbiota in the recurrent group. Logistic regression analysis showed that the composition of stones, the high abundances of *P. dorei* and *Fusobacterium necrogenes* were potential risk factors for recurrent choledocholithiasis, and a nomogram based on these factors demonstrated high accuracy and excellent calibration. This study has identified potential risk factors for recurrent choledocholithiasis and built a nomogram that can well predict recurrent risks for patients who undergo cholecystectomy, which might serve as a useful tool for patients’ stratification and post-surgery management.

**IMPORTANCE:**

This study identifies specific gut microbiome signatures of gallstone patients and indicates that reduced diversity and high abundances of *Phocaeicola dorei* and *Fusobacterium necrogenes* might be potential predictors for choledocholithiasis recurrence after cholecystectomy. By demonstrating a link between gut microbiota composition and post-surgical recurrence risk, it advances our understanding beyond simple association with predictive capability. The development of a nomogram incorporating these microbial markers provides a novel, clinically applicable tool for accurate risk stratification of patients undergoing cholecystectomy, therefore bridging the gap between microbial ecology and clinical practice. The significance of this study lies in that it aims to address a critical unmet need in gallstone disease management by offering a more cost-effective and non-invasive tool to improve long-term patient outcomes and pave the way for microbiome-targeted interventions.

**CLINICAL TRIALS:**

This study is registered with Chinese Clinical Trial Registry Center as ChiCTR2400090232.

## INTRODUCTION

Cholelithiasis, also named gallstone disease, is a common benign digestive disease with a prevalence ranging between 6.5% and 7.6% in China (1, 2). Gallstone disease can be asymptomatic or symptomatic. The most common symptom is recurrent upper abdominal pain and biliary indigestion. According to *Consensus on the Surgical Management of Benign Gallbladder Diseases (2021 edition*) (3), laparoscopic cholecystectomy is the first-line surgical treatment recommended for symptomatic cholelithiasis, and asymptomatic cholelithiasis is also an indication for surgery. Cholecystectomy, however, may cause short-term and long-term side effects, including biliary injury, post-cholecystectomy syndrome, diarrhea, or even cancers (4). In addition, though the surgical method can remove up to 95% of stones, the incidence of recurrent choledocholithiasis remains 4%–25% (5–7). Some researchers suggested that post-cholecystectomy patients undergo routine clinical follow-up procedures to detect recurrent bile duct stones early (8). But these repeated tests and hospital visits are time-consuming and cause economic burdens to patients. Therefore, a prompt recurrent risk assessment at admission and after cholecystectomy can further stratify patients to undergo routine follow-ups. Besides, cholecystectomy is not a once-and-for-all solution for gallstones. Post-surgery management, including ursodeoxycholic acid, lifestyle, and diet, might prevent gallstone formation to lower the recurrent rate and prevent biliary-related complications (9, 10).

Many factors may contribute to the formation and recurrence of stones. In fact, a string of studies has identified risk factors for recurrent common bile duct stones (11, 12); however, little is known regarding how these factors can be utilized to instruct clinical practice. According to these studies, several first-episode stone-related factors can influence the recurrence of common bile duct stones, including the size and number of stones, composition and properties of stones (i.e., pigment stones), and the clinical characteristics of patients (7, 13, 14). With the advent of high-throughput sequencing techniques, the microbiota has also been identified as a risk factor for stone formation and recurrence. For example, Liu et al. (15) have revealed that the bile microbiome can modulate choledocholithiasis onset and recurrence by altering metabolism. And Li et al. (16) reported that *Fusobacterium* and *Neisseria* in the bile were associated with recurrent cases. In addition, studies have accumulated that the gut microbiota was also involved in the formation of gallstones. For example, Keren et al. (17) found decreased gut microbial diversity, a reduction in *Roseburia,* and an enrichment of *Oscillospira* in gallstone patients. Hu et al. (18) revealed the role and mechanism of gut *Desulfovibrionales* in cholesterol gallstone formation. Interestingly, studies showed a significant difference in the bile microbiota compositions between cholesterol and pigment stones (19). But studies on the gut microbiota differences between these two types of gallstones are scarce. Since the pigment stones were reported to be associated with choledocholithiasis recurrence, and the gut microbiota may serve as a better biomarker for its convenience in sample collection, studies that fully illustrate the gut microbiome of pigment stones and recurrent choledocholithiasis might pave the way for developing a novel risk stratification tool and post-surgery management options.

In the present study, we first investigated the gut microbiota variances between gallstone patients and healthy controls, then delved into the gut microbiota differences between cholesterol stones and pigment stones. After laparoscopic cholecystectomy, patients were carefully followed up, and recurrent common bile duct stone cases were recorded. We further screened the potential microbiologic biomarkers for recurrent cases. Finally, a recurrent risk predicting model was built based on critical clinical data and the feature microbiota in recurrent cases to support clinical decision-making.

## MATERIALS AND METHODS

### Study design and specimen collection

From September 2020 to June 2021, patients with symptomatic gallstone disease were screened and enrolled into the Disease group in the Department of Hepatobiliary Surgery at Quanzhou First Hospital. The inclusion criteria were as follows: (i) aged 40–80 years, (ii) scheduled for laparoscopic cholecystectomy, and (iii) informed consent. The exclusion criteria were as follows: (i) suspected of gallbladder malignancy, (ii) antibiotics or probiotics usage within 1 month, and (iii) comorbid intestinal or bowel diseases. Meanwhile, age- and sex-matched individuals who were ruled out of gallstone disease by imaging were enrolled into the Control group. All patients in the Disease group received laparoscopic cholecystectomy, while those comorbid with choledocholithiasis further received endoscopic retrograde cholangiopancreatography (ERCP) or laparoscopic choledochotomy (LCD) accordingly to achieve complete stone clearance. Clinical characteristics and laboratory indicators were recorded. All participants were taught to collect fecal samples with an exclusive stool collector (Biotecan, Shanghai, China) before any treatment, which were stored immediately at −80°C and shipped with sufficient dry ice for microbiological analysis. Written informed consents were obtained from all participants before sample collection. The study protocol was approved by the Ethics Committee of Quan Zhou First Hospital (#2020252). This study was registered on the Chinese Clinical Trial Registry Center (https://www.chictr.org.cn/) (ID: ChiCTR2400090232) and was reported in compliance with the Strengthening the Reporting of Observational Studies in Epidemiology (STROBE) (20) and Strengthening the Reporting Of Cohort Studies in Surgery (STROCSS) criteria (21).

### Bacterial DNA extraction and 16S rDNA sequencing

The microbiological analysis was carried out in Shanghai Biotecan Pharmaceuticals Co., Ltd (Shanghai, China). Upon arrival at the laboratory, the samples were thawed on ice and then processed according to standard procedures as described elsewhere (22). Briefly, the total bacterial genomic DNA was first extracted with the PowerMax DNA isolation kit (MoBio Laboratories, Carlsbad, CA, USA), followed by being quantified and qualified with gel electrophoresis and a NanoDrop ND-1000 spectrophotometer (Thermo Fisher Scientific, Waltham, MA, USA). Then, polymerase chain reaction (PCR) was performed in a PCR system to amplify the V4 region of the bacterial 16S rDNA, using the forward primers 341F (5′-CCTAYGGGRBGCASCAG-3′) and the reverse primer 806R (5′-GGACTACHVGGGTWTCTAAT-3′). Then, PCR amplicons were purified and quantified, followed by being pooled in equimolar quantities. Paired-end 2 × 150 bp sequencing was performed on the Illumina NovaSeq 6000 platform.

### Bioinformatic analysis

First, match raw sequencing reads to the barcodes and assign them to the corresponding samples. Then, filter the raw reads following the below criteria: (i) sequences under 150 bp were removed, (ii) sequences with average Phred scores under 20 were removed, (iii) discard sequences that contained ambiguous bases, and (iv) discard sequences that contained mononucleotide repeats over 8 bp. Next, assemble paired-end reads with Vsearch v2.4.4, and assign unique sequences to operational taxonomic units (OTUs) with mothur (v1.39.5). Then, the Greengenes2 database was used to verify the taxonomy of these OTUs and subsequently generate an OTU table recording the abundance of each OTU in a single sample. The Quantitative Insights into Microbial Ecology (QIIME2, v2023.2.0) and R packages (v3.2.0) were applied to calculate microbiome diversity indices, including the α-diversity (the Chao1, ACE, Shannon, observed species, and Simpson indexes), and the β-diversity indices, which were visualized with the principal component analysis (PCA), principal coordinate analysis (PCoA), and non-metric multi-dimensional scaling (NMDS). Moreover, the R stats package was applied for the linear discriminant analysis (LDA) effect size analysis (LEfSe), with the cutoff value of the absolute LDA score (log10) >2.0 and *P* < 0.05. In addition, ANCOM-BC2 (23) and Deseq2 (24) were also applied for differential abundance analysis. Finally, PICRUSt2 (https://github.com/picrust/picrust2/) was applied to predict the microbial functions and enrich the functional pathways with the Kyoto Encyclopedia of Genes and Genomes (KEGG) database.

### Statistical analysis

The clinical prediction model was built using R version 4.4.0 (2024-04-24), along with Zstats 1.0 (https://www.zstats.cn/). First, the data set containing clinical variables and the feature microbiota that were potentially associated with gallstones recurrence was uploaded on the platform and divided into a training set and a validation set at 7:3 with a random seed of 469. Then, univariable logistic regression analysis was performed on the clinical parameters and the abundances of *P. dorei* and *F. necrogenes*. The variables with *P* < 0.1 were entered into multivariable analysis to identify the risk factors for gallstone recurrence. Next, a nomogram for gallstones recurrent risk was constructed based on the multivariable analysis results with *P* < 0.05. Finally, the performance of the nomogram was assessed by receiver operating characteristic curve (ROC), calibration plot with Hosmer and Lemeshow goodness of fit (GOF) test, and decision curve analysis (DCA) performed with the R “dcurves” package. In addition, the nomogram was subjected to 1,000 bootstrap resamples for internal validation to assess its predictive accuracy.

Normally distributed continuous data were displayed as mean ± SD, while the category variables as number (%). Student’s t-test or one-way ANOVA was used in comparisons of normal distribution data, while Kruskal-Wallis tests or Mann-Whitney *U* test were applied in comparisons of non-normal distribution data. For the group comparisons of microbial data, the Wilcoxon rank-sum test and Permutational multivariate analysis of variance (PERMANOVA) were used. Spearman’s rank correlation analysis was performed via SPSS (v27.0). All *P-*values were two-sided, and *P* < 0.05 was considered statistically significant.

## RESULTS

### Clinical characteristics of all participants

**Fig 1:**
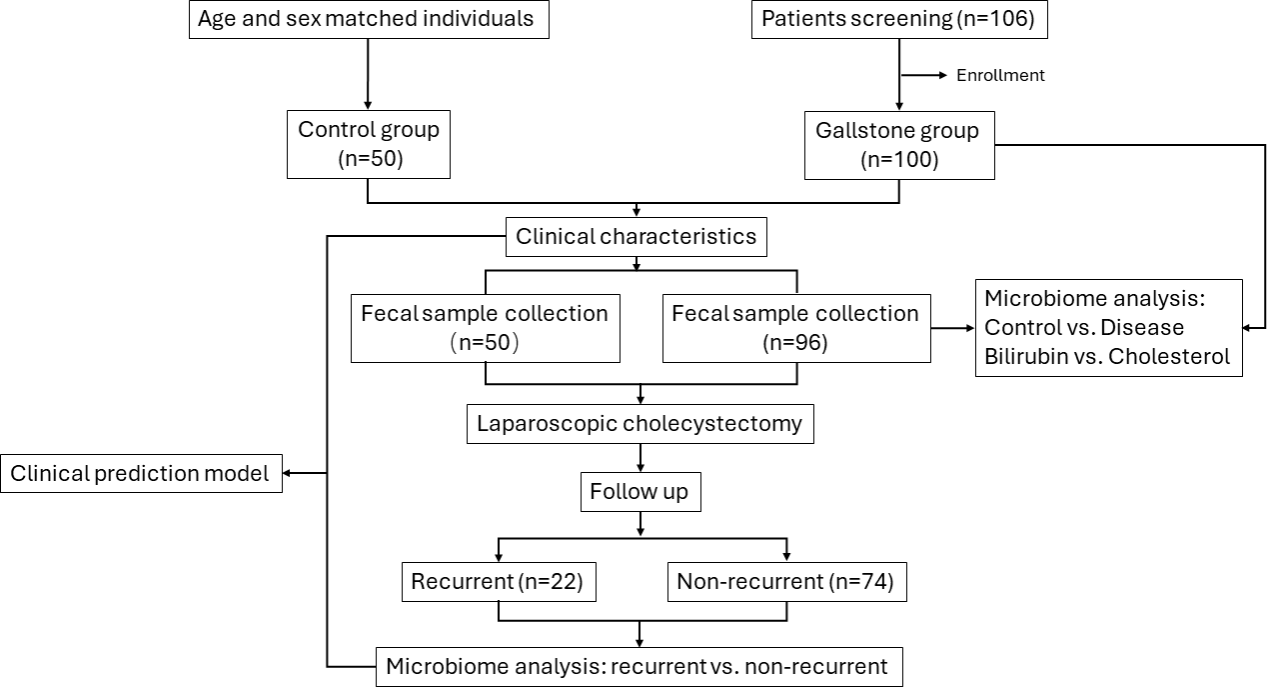
The flowchart of the study design. Four patients were excluded from microbiome analysis due to poor quality of fecal samples.

A total of 100 patients and 50 gallstone-free individuals were enrolled in this study. The age and sex were comparable between the two groups. The average body mass index (BMI) of the Control group was slightly lower than the Disease group, but with no significant difference (*P* > 0.05). All patients received laparoscopic cholecystectomy, while 16 (16.67%) patients received additional ERCP or LCD to remove common bile duct stones. The detailed clinical characteristics are shown in Table 1 and File S1. Within 1 year after surgery, 42 (43.75%) patients presented with diarrhea, and four (4.17%) patients were reported to have biliary pain. After a median follow-up time of 39.4 months, 22 (22.91%) patients had recurrent bile duct stones, with a median recurrent time being 17.5 months post-surgery. There was no correlation between the recurrent bile duct stones and pre-cholecystectomy bile duct stones (*P* > 0.05). The flowchart of this study design is displayed in Fig. 1.

**TABLE 1:** Clinical characteristics of all participants^*a*^

Characteristic	Control group (*n* = 50)	Gallstone group (*n* = 100)	*P*-value
Age (mean ± SD)	56.86 ± 10.47	55.55 ± 12.12	0.518
Sex (*n*, %)			
Male	28 (56.00%)	45 (46.88%)	0.295
Female	22 (44.00%)	51 (53.13%)	
BMI (mean ± SD)	22.69 ± 2.78	23.77 ± 3.85	0.054
Smoking habits (*n*, %)			
Current smoker	2 (4.00%)	9 (9.38%)	0.502
Never smoke	45 (90.00%)	82 (85.42%)	
Unknown	3 (6.00%)	5 (5.21%)	
Gallstone composition (*n*, %)			
Pigment	/^*b*^	29 (30.31%)	
Cholesterol	/	58 (60.42%)	
Not documented	/	9 (9.38%)	
Comorbidities (*n*, %)			
Infection	/	58 (60.42%)	
Common bile duct stones	/	16 (16.67%)	
Pathologic evaluation (*n*, %)			
Acute cholecystitis	/	5 (5.21%)	
Chronic cholecystitis	/	75 (78.13%)	
Gallbladder polyp	/	1 (1.04%)	
Gallbladder adenomyomatosis	/	2 (2.08%)	
Gallbladder surface erosion	/	4 (4.17%)	
Presence of RA sinus	/	2 (2.08%)	
Not documented	/	7 (7.29%)	
Post-cholecystectomy complications (*n*, %)			
Diarrhea	/	42 (43.75%)	
Abdominal pain	/	4 (4.17%)	
Laboratory tests (median, range)			
WBC (10^9^/L)	/	6.18 (3.26–17.15)	
TBil (μmol/L)	/	14.75 (6.80–109.10)	
DBiL (μmol/L)	/	3.00 (1.00–51.80)	
ALP (U/L)	/	86.50 (1.41–777.00)	
ALT (U/L)	/	23.50 (13.00–424.00)	

^*a*^ BMI: body mass index; RA: Rokitansky-Aschoff; WBC: white blood cell counts; TBil: total bilirubin; DBiL: direct bilirubin; ALP: alkaline phosphatase; ALT: alanine transaminase.

^*b*^ "/,” not applicable.

### Gut microbiome features of gallstone patients compared to healthy adults

**Fig 2:**
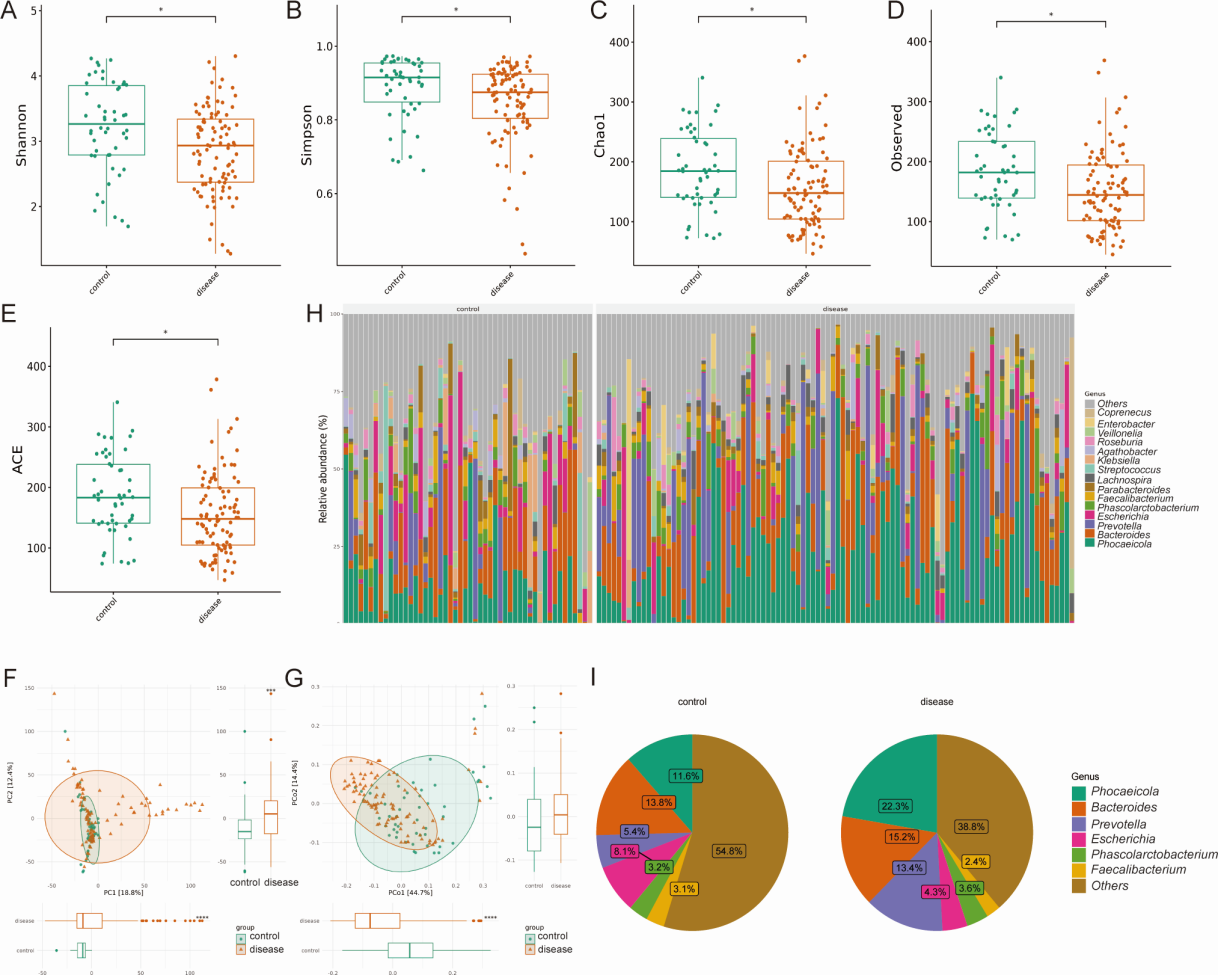
Differences in gut microbiota diversity and taxa composition between the Control group and the Gallstone group. Differences in α-diversity between two groups regarding Shannon index (**A**), Simpson index (**B**), Chao1 index (**C**), Observed species (**D**), and ACE index (**E**). Differences in β-diversity between the two groups regarding the unweighted unifrac PCA plot (**F**) and the unweighted unifrac PCoA plot (**G**). Bar plot (**H**) and pie plot (**I**) of the taxa distribution of the two groups at the genus level.

**Fig 3:**
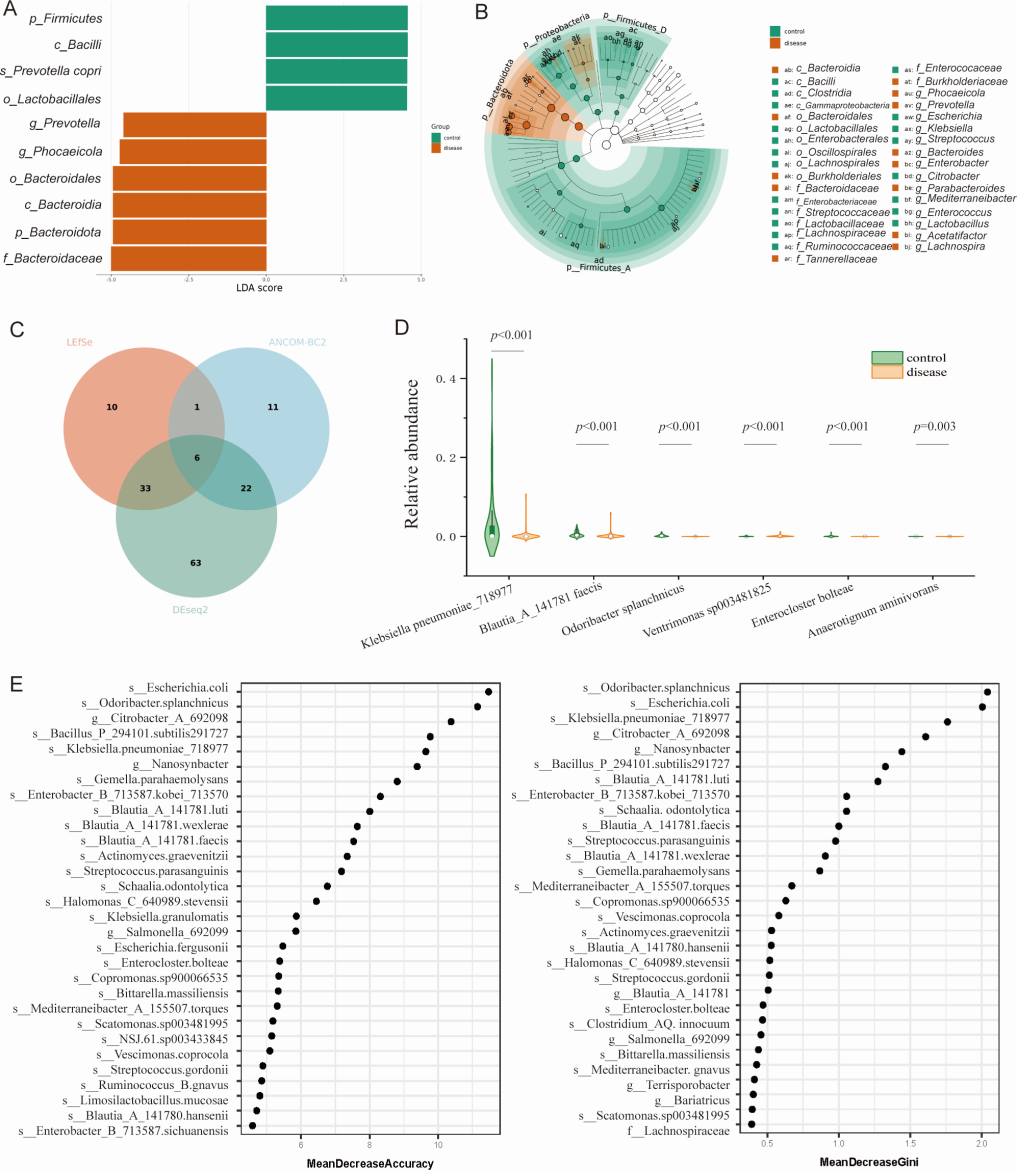
Feature microbiota and functional prediction of gallstone patients. LEfSe analysis results (**A, B**) showed that phylum Bacteroidota and genus Phocaeicola were the feature microbiota in gallstone patients. (C) Venn diagram of differential abundance analysis results by LEfSe, ANCOM-BC2, and DEseq2 analyses. (D) Relative abundance of the six differentially abundant taxa between the two groups. (E) Random forest analysis of the feature microbiota.

All the α-diversity indexes, including Shannon, Simpson, Chao1, ACE, and observed species in the Disease group were significantly lower than those of the Control group (all *P* < 0.05) (Fig. 2A through E). Meanwhile, there was a significant difference in β-diversity between the two groups, either in PCA or PCoA analysis (Fig. 2F and G). The bar plot of microbiota distribution across samples at the genus level is found in Fig. 2H. The top three dominant genera in the Disease group were *Phocaeicola* (22.3%), *Bacteroides* (15.2%), and *Prevotella* (13.4%), while in the Control group were *Phocaeicola* (11.6%), *Bacteroides* (13.8%), and *Escherichia* (8.1%) (Fig. 2I). LEfSe analysis showed that phylum *Bacteroidota* was dominant in the disease group (LDA score = 4.94, *P* < 0.001), while the phylum *Firmicutes* dominated in the Control group (LDA score = 4.56, *P* < 0.001); at genus level, the feature microbiota in the gallstone group were *Phocaeicola* (LDA score = 4.72, *P* < 0.01) (Fig. 3A and B) (File S2). Further differential abundance analysis with LEfSe, ANCOM-BC2, and DEseq2 found six shared species that were differently abundant in the Control and Disease groups, that is, *Klebsiella pneumoniae* (3.75% vs. 0.16%, *P* < 0.001), *Blautia faecis* (0.54% vs. 0.16%, *P* < 0.001), *Odoribacter splanchnicus* (0.19% vs. 0.00%, *P* < 0.001), *Ventrimonas* (0.03% vs. 0.11%, *P* < 0.001), *Enterocloster bolteae* (0.05% vs. 0.00%, *P* < 0.001), and *Anaerotignum aminivorans* (0.00% vs. 0.00%, *P* = 0.003) (Fig. 3C and D). Random forest analysis revealed that species *O. splanchnicus* could well differentiate two groups with Mean Decrease Accuracy being 11 and Mean Decrease Gini being 2 (Fig. 3E). Further functional prediction showed that the microbiome of the gallstone patients mainly enriched on metabolism pathways, including glycan, amino acid, and cofactors metabolisms, while that of the control group enriched on membrane transport (Fig. S1).

### Gut microbiome features of recurrent patients compared to non-recurrent patients

**Fig 4:**
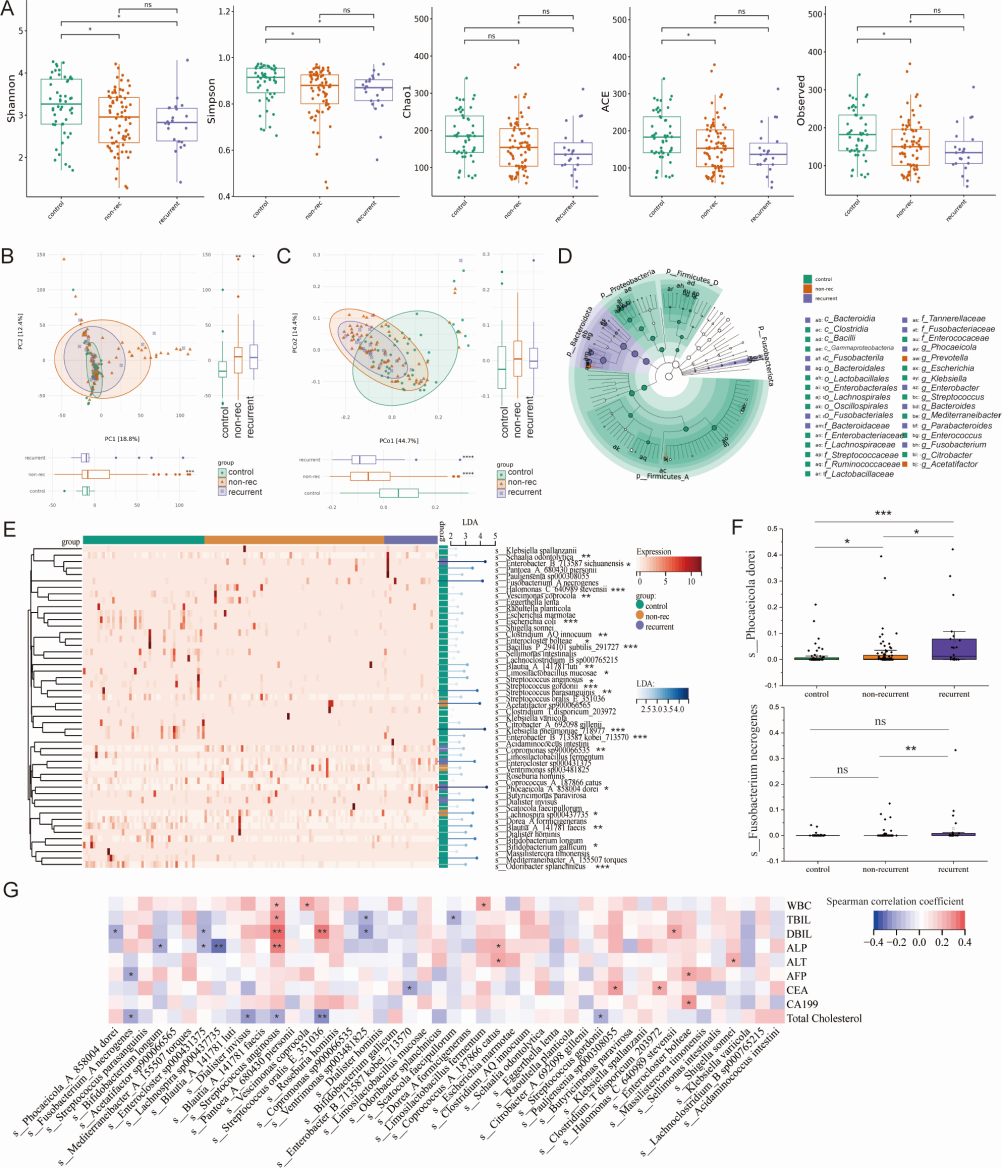
Gut microbiome characteristics of recurrent gallstone patients. (A) Differences in α-diversity among the control, recurrent, and non-recurrent groups. (B) Differences in weighted unifrac PCA analysis. (C) Differences in weighted unifrac PCoA analysis. (D) LEfSe analysis result showed that the recurrent patients were featured by phylum Bacteroidota and phylum Fusobacteriota. (E) Heatmap of the top 50 differentially abundant species with LDA score >2. (F) Bar plot of the abundance of the species *Phocaeicola dorei* and the species *Fusobacterium necrogenes*. (G) Spearman correlation analysis of the differentially abundant species and clinical laboratory indicators.

Although there was no significant difference in α-diversity between recurrent (*n* = 22) and non-recurrent (*n* = 74) groups, the Chao1 index of the recurrent group was remarkably lower than the control group (*P* < 0.05), while no significant difference was observed between the non-recurrent and the control groups (Fig. 4A). As visualized in PCA and PCoA clustering (Fig. 4B and C), both recurrent and non-recurrent groups differed remarkably in β-diversity from the control group, but no obvious difference was observed between them. As demonstrated in the cladogram (Fig. 4D), phylum *Bacteroidota* and phylum *Fusobacteriota* were dominant in the recurrent group. LEfSe analysis also identified 50 species (LDA score > 2) that were differentially abundant in the control, recurrent, and non-recurrent groups, which identified *P. dorei* as a feature microbiota in the recurrent group (LDA score = 4.2, *P* < 0.05) (Fig. 4E) (File S3). As displayed in Fig. 4F, the abundance of *P. dorei* in the recurrent group was significantly higher than that of the non-recurrent group (*P* < 0.05) and the control group (*P* < 0.001). Also, the abundance of *Fusobacterium necrogenes* in the recurrent group was significantly higher than that of the non-recurrent group (*P* < 0.01), but no significance was observed compared with the control group (*P* > 0.05). Further Spearman correlation analysis between these species and clinical laboratory indicators showed that the abundance of *P. dorei* was negatively related to the blood direct bilirubin (DBiL) (*r* = −0.223, *P* < 0.05), while *Streptococcus anginosus* (*r* = 0.339, *P* < 0.01) and *Streptococcus oralis* (*r* = 0.315, *P* < 0.01) were positively correlated with DBiL (Fig. 4G).

### Gut microbiome features of pigment stones and the association with blood bilirubin

**Fig 5:**
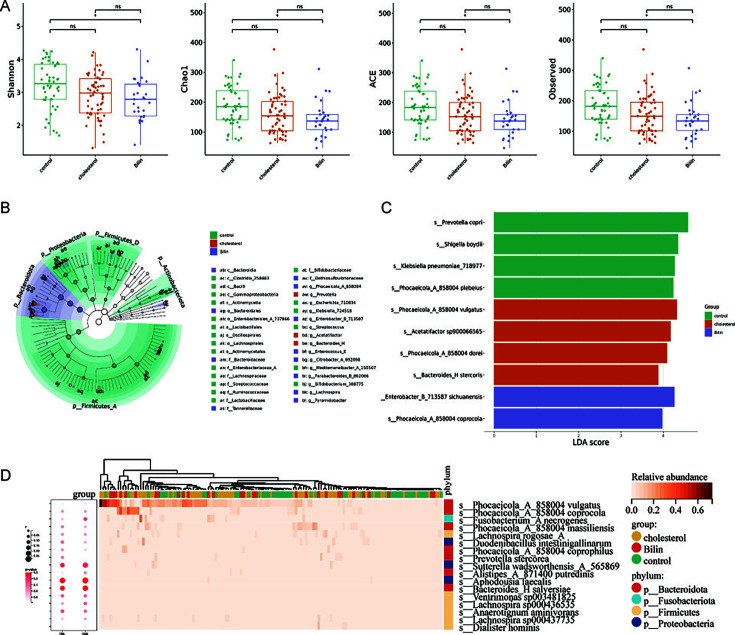
Gut microbiome characteristics of pigment and cholesterol gallstones. (A) Differences in α-diversity among the control, pigment, and cholesterol gallstone groups. (B) Cladogram of the featured microbiota. (C) LEfSe analysis result showed that *P. dorei* and *P. vulgatus* were enriched in the cholesterol gallstone group. (D) Heatmap of the differentially abundant species and their correlations with blood bilirubin.

To investigate the potential association between gut microbiota and the composition of gallstones, patients were subdivided into the pigment group (*n* = 29) and the cholesterol group (*n* = 58) depending on their gallstone major composition. It turned out that the α-diversity indexes, including Shannon, Chao1, ACE, and observed species, were significantly lower in the pigment group than in the control group (all *P* < 0.05) (Fig. 5A). As displayed in the cladogram, phylum Bacteroidota also dominated in the pigment group (Fig. 5B). LEfSe analysis identified many feature species in pigment group, including *P. coprocola* (LDA score = 3.9, *P* > 0.05) and *Enterobacter sichuanensis* (LDA score = 4.3, *P* < 0.05) (Fig. 5C) (File S4). Differential abundance analysis with the ANCOM-BC2 tool filtered out 17 species to be differentially abundant in the pigment group with a threshold of log2(Fold Change)>1 and *P* < 0.05. The heatmap of these species and their correlations with blood bilirubin is shown in Fig. 5D. Among these species, *Alistipes putredinis* was negatively correlated with TBiL (*r* = −0.254, *P* < 0.05) and DBiL (*r* = −0.279, *P* < 0.01), while *Prevotella stercorea* was positively correlated with DBiL (*r* = 0.243, *P* < 0.05).

### Clinical prediction model for recurrent choledocholithiasis risk

**Fig 6:**
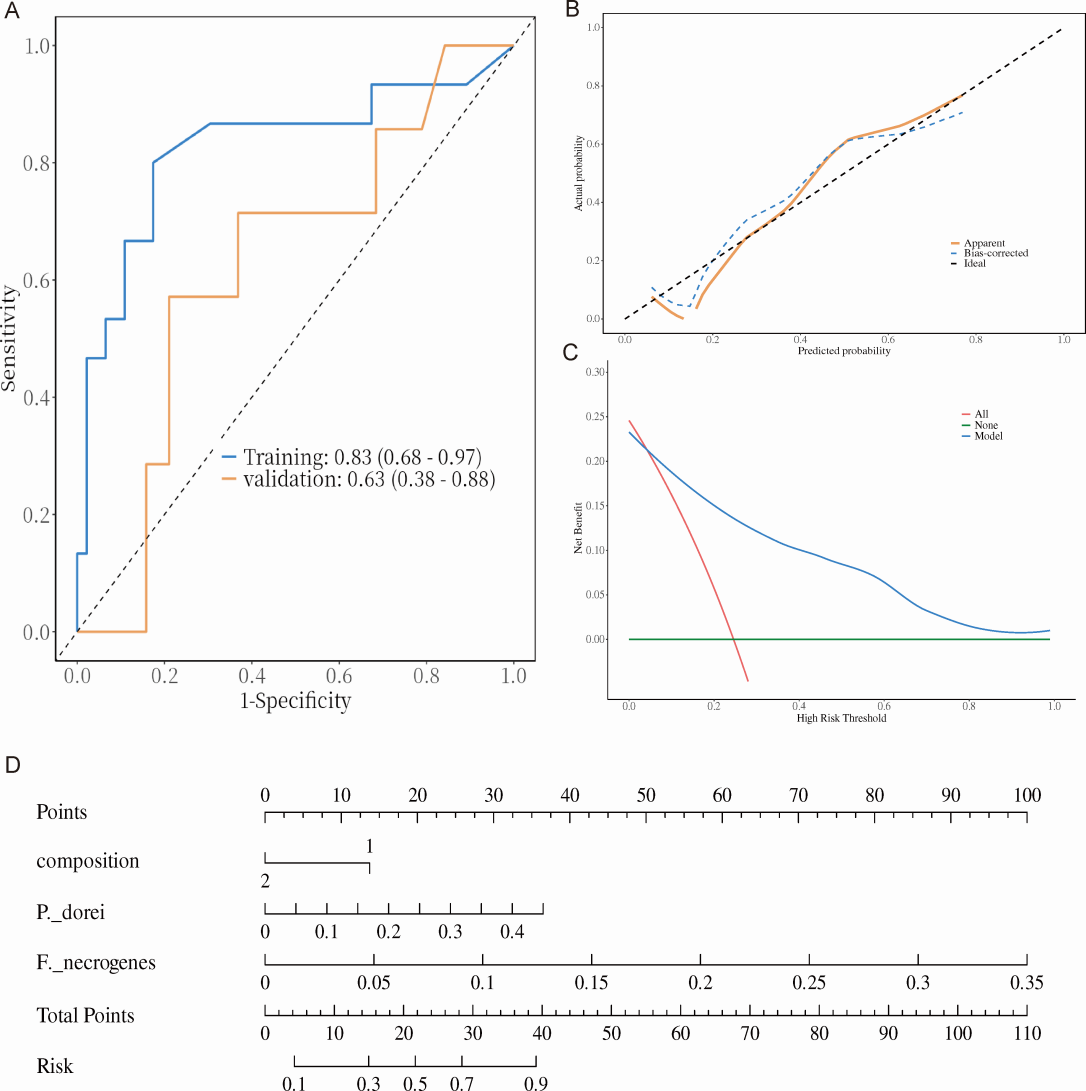
Clinical prediction model built by logistic regression analysis. (A) ROC curves of the training set and validation set. (B) Calibration performance of the clinical prediction model. (C) Decision curve analysis of the clinical prediction model. (D) Nomogram of the clinical prediction model. Composition 1 refers to pigment stones; composition 2 refers to cholesterol stones.

According to the univariable and multivariable logistic regression results (Table 2), three variables, including gallstone composition, *P. dorei,* and *F. necrogenes,* entered the clinical prediction model with the VIF values ≈ 1, indicating that no collinearity existed between these variables. The regression residuals plot is found in Fig. S2. The discrimination performance of the model was determined by the area under curve (AUC) of the ROC curve, which showed that the training set is 0.83 (95% confidence interval [CI]: 0.68–0.97) and the validation set is 0.63 (95% CI: 0.38–0.88) (Fig. 6A). The confusion matrix of the model is displayed in Table 3. The calibration performance plot showed a good predictive accuracy between the actual probability and predicted probability with *P* = 0.278 in the training set and *P* = 0.137 in the validation set (H-L GOF test) (Fig. 6B). As displayed in Fig. 6C, the predicting model could lead to a higher net benefit than treat for none and treat for all under the threshold of 0.05-1.0. In summary, the nomogram for predicting recurrent choledocholithiasis risk (Fig. 6D) could serve as a realistic clinical tool for patients who underwent laparoscopic cholecystectomy.

**TABLE 2:** Logistics analysis of predictors for recurrent cases^*a*^

Variables	Univariate					Multivariate				
	β	SE	*Z*	*P*	OR (95% CI)	β	SE	*Z*	*P*	OR (95% CI)
Composition										
1					1 (reference)					1 (reference)
2	−1.29	0.61	−2.09	0.036	0.28 (0.08–0.92)	−1.73	0.77	−2.26	0.024	0.18 (0.04–0.80)
*P. dorei*	8.05	3.77	2.14	0.033	3,137.05 (1.94–5.07E6)	10.21	4.09	2.50	0.012	27,270.81 (9.06–8.21E7)
*F. necrogenes*	28.68	14.06	2.04	0.041	2.85E12 (3.09–2.64E24)	35.98	15.73	2.29	0.022	4.21E15 (172.93–1.02E28)

^*a*^ OR: odds ratio, CI: confidence interval. Composition 1 refers to pigment stones; composition 2 refers to cholesterol stones.

**TABLE 3:** Confusion matrix of training set and validation set^*a*^

Set	AUC (95% CI)	Accuracy (95% CI)	Sensitivity (95% CI)	Specificity (95% CI)	PPV (95% CI)	NPV (95% CI)	Cutoff
Training	0.83 (0.68–0.97)	0.80 (0.68–0.89)	0.83 (0.72–0.94)	0.73 (0.51–0.96)	0.90 (0.82–0.99)	0.58 (0.36–0.80)	0.269
Test	0.63 (0.38–0.88)	0.69 (0.48–0.86)	0.74 (0.54–0.93)	0.57 (0.20–0.94)	0.82 (0.64–1.00)	0.44 (0.12–0.77)	0.269

^*a*^ AUC: area under curve; CI: confidence interval; PPV: positive predictive value; NPV: negative predictive value.

## DISCUSSION

Gut microbiota is recorded to be associated with maintaining healthy conditions or promoting disease development. Accumulating evidence showed that gut microbiome dysbiosis might lead to gallstone genesis via disturbing bile acid homeostasis (25). Studies found that the gut and biliary tract microbiome composition and diversity varied significantly in gallstone patients and healthy subjects (17, 26, 27), and cholecystectomy might also reduce microbiota diversity and lead to microbiome dysbiosis (28). Consistent with these reports, the present study also showed that the gut microbiome diversity was significantly reduced in the gallstone group when compared to controls, with genera *Phocaeicola* and *Prevotella* being remarkably abundant in the disease group. *Phocaeicola* is a gram-negative genus in the phylum of *Bacteroidota*. To date, a total of 14 species have been classified, including *P. vulgatus, P. barnesiae, P. caecicola, P. caecigallinarum, P. chincillae, P. coprocola, P. coprophilus, P. dorei, P. gallinaceum, P. massiliensis, P. paurosaccharolyticus, P. plebeius, P. salanitronis,* and *P. sartorii*. Among these, *P. dorei* was reported to be positively correlated with unconjugated bile acids (29). In the present study, we found that *P. dorei* was more abundant in the recurrent group than in the non-recurrent (*P* < 0.05) and control group (*P* < 0.001). Besides, there was a negative relationship between the abundance of *P. dorei* and DBil, indicating a potential role in regulating the composition of bile. *In vitro* study showed that *P. dorei* could reduce cholesterol and bile salt hydrolase activity (30). In addition, a Mendelian randomization study revealed a causal relationship between *P. dorei* and blood lipids (31). Since a low level of lipids might increase risks for gallstones (32), these studies indicate a potential role of *P*. dorei in the genesis of gallstones.

Gallstones can be categorized into cholesterol stones and pigment stones according to the stones’ major constituents (33). Only a few studies have investigated the differences in the microbiome between cholesterol and pigment stones. Georgescu et al. (34) found that the biodiversity of gut microbiome in cholesterol stones decreased when compared to pigment stones, while Zhang et al. (19) revealed no significant difference in bile microbiota between the two stone types. In the present study, although the α-diversity of pigment stones was significantly lower when compared to controls, no significant difference was observed between cholesterol and pigment stones in gut microbiota. LEfSe analysis showed that *P. dorei* and *P. vulgatus* were abundant in the cholesterol group, while *P. coprocola* was abundant in the pigment group. *P. vulgatus* was involved with carbohydrate metabolism and was associated with colitis, atherosclerosis, and type 2 diabetes (35, 36). Recently, research also reported that *P. vulgatus* could reduce histone acetylation of genes involved in lipid metabolism to protect against metabolic dysfunction-associated steatotic liver disease (MASLD) (37). Therefore, both *P. dorei* and *P. vulgatus* are associated with lipid metabolism and can be potential biomarkers for cholesterol stones.

The biological risk factors for recurrent common bile duct stones focused on bile and gut bacteria and their products, such as anaerobes, exogenous β-glucuronidase, and insoluble free bile acids (38, 39). Li et al. revealed that *Fusobacterium* and *Neisseria* were abundant while Lactobacillus was absent in the bile of recurrent patients (16). Meanwhile, Liu et al. reported the predictive value of *Actinomycetes* for common bile duct stone recurrences (15). In the present study, *P. dorei* and *F. necrogenes* were abundant in recurrent cases, and a clinical prediction model based on biological and clinical indexes displayed a good prediction effect (AUC = 0.83). Besides, the nomogram built thereafter can serve as a useful tool for clinicians’ decision-making and patients’ management. In this nomogram, the pigment gallstones, high abundances of *P. dorei* and *F. necrogenes* contributed to recurrent risks to different degrees. Similar nomograms have been built for other conditions, such as acute biliary pancreatitis (40), active tuberculosis (41), invasive pulmonary adenocarcinoma (42), and so on. However, to our knowledge, this is the first nomogram for predicting post-cholecystectomy stone recurrences among gallstone patients.

Limitations are inevitable in this study. First, this study only investigated the pre-surgery gut microbiome features and the gut microbiota dynamic changes after surgery. Besides, the clinical model was internally validated with a relatively low AUC value in the testing cohort. The residual plot of the model suggests that there might be a bias in the model. An improved model should be built and demand external validation. Therefore, repeated testing of gut microbiota and a multi-center cohort need to be built into the coming studies.

### Conclusions

This study revealed a different gut microbiome between gallstone patients and controls and identified recurrence-associated biological factors for those who underwent cholecystectomy. The novel nomogram for predicting the risk of recurrent choledocholithiasis after laparoscopic cholecystectomy is highly accurate and exhibits excellent calibration, which might serve as a useful tool for post-surgery management.

## Data Availability

Raw sequence data can be obtained from NCBI (https://www.ncbi.nlm.nih.gov/) at SRA accession number PRJNA1164942. Other data that support the findings of this study are available from the corresponding author upon reasonable request.

## AFTER EPUB

*[This article was published on 25 July 2025 with errors in Fig. 5 and the affiliations. The figure was corrected on 30 July 2025, and the affiliations were corrected in the current version, posted on 31 July 2025.]*

## SUPPLEMENTAL MATERIAL

File S1 msystems.01760-24-s0001.xlsx Clinical characteristics.

File S2 msystems.01760-24-s0002.xls LDA scores in gallstone group.

File S3 msystems.01760-24-s0003.xls LDA scores in recurrent group.

File S4 msystems.01760-24-s0004.xls LDA scores in pigment group.

Figure S1 msystems.01760-24-s0005.tif Predicted metabolic fuctions of microbiota.

Figure S2 msystems.01760-24-s0006.tiff Regression residuals.

Legends msystems.01760-24-s0007.docx Legends for supplemental figures and files.

ASM does not own the copyrights to Supplemental Material that may be linked to, or accessed through, an article. The authors have granted ASM a non-exclusive, world-wide license to publish the Supplemental Material files. Please contact the corresponding author directly for reuse.
