# Supplementary figures and images for: The gut microbiota features and the application value in predicting recurrent risks for gallstone patients who underwent laparoscopic cholecystectomy
Source: mSystems. 2025 Jul 25;10(8):e01760-24. doi: 10.1128/msystems.01760-24 (PMC12363225; doi:10.1128/msystems.01760-24)

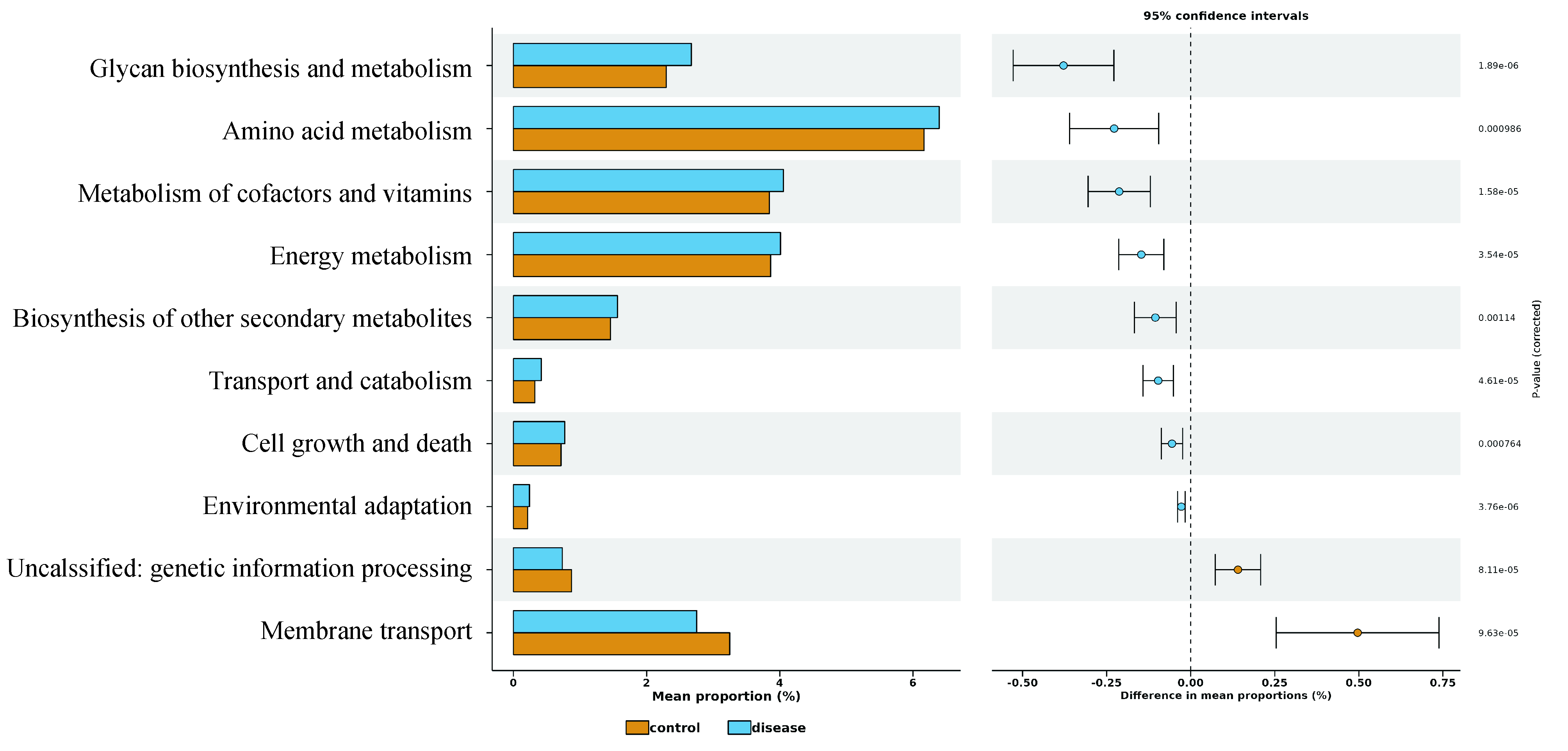

Supplement: Figure S1 — Predicted metabolic fuctions of microbiota. [file msystems.01760-24-s0005.tif]

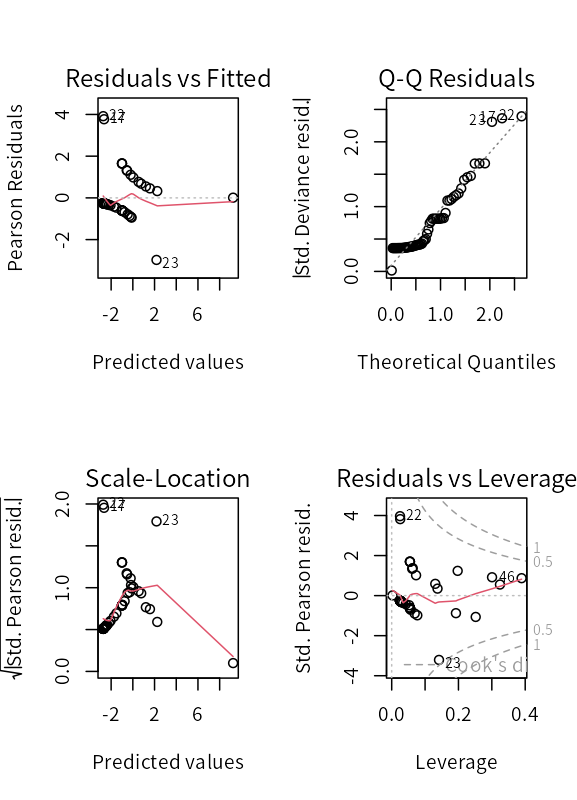

Supplement: Figure S2 — Regression residuals. [file msystems.01760-24-s0006.tiff]
