# Supplementary material for: The gut microbiota features and the application value in predicting recurrent risks for gallstone patients who underwent laparoscopic cholecystectomy
Source: mSystems. 2025 Jul 25;10(8):e01760-24. doi: 10.1128/msystems.01760-24 (PMC12363225; doi:10.1128/msystems.01760-24)
Supplement: Legends — for supplemental figures and files. [file msystems.01760-24-s0007.docx]

Figure S1. Functional prediction of the feature microbiota. The gut microbiome of the gallstone patients mainly enriched on metabolism pathways, while that of the control group enriched on membrane transport.

Figure S2. Residual plot of the regression model. The Residuals vs Fitted, Normal Q-Q, Scale-Location, and Residuals vs Leverage plots showed that there were patterns in the residuals.

Supplementary file 1. Clinical characteristics of participants.

Supplementary file 2. Featured microbiota of gallstone group with LDA scores and p-values.

Supplementary file 3. Featured microbiota of recurrent group with LDA scores and p-values.

Supplementary file 4. Featured microbiota of pigment stone group (bilin) with LDA scores and p-values.
